# Remarks on Cancer of the Breast

**Published:** 1873-11

**Authors:** 


We select the following from the discussion concerning Cancer of the Breast, at a recent meeting of the Medical Library and Journal Association of New York.

DR. FORDYCE BARKER: Mr. President, my apology for departing from my usual rule with regard to surgical questions and operations is, that I may perhaps suggest some new fields for inquiry and observation, and perhaps bring out some new ideas in the course of the discussion by these suggestions. In regard to surgery, I am no expert. I do not pretend even to interfere with it, and it is therefore somewhat embarrassing to speak upon a subject which really belongs to the surgical department. I have, however, had occasion to study the subject of cancer, with great interest, and perhaps with a large experience, and have, therefore, for many years taken every pains to inform myself with regard to the progress of science, and have felt an interest in its bearing upon the question of its manifestation in the form in which it occurs secondarily, which in its most frequent form is that of cancer of the breast.

In alluding to certain points in connection with the general subject, I will refer to one or two cases in connection with my own personal experience. Previous to my coming to this city, I was obliged to practice more or less in general surgery, and in the course of that time I was called upon to amputate the breast thirteen times, for what I supposed to be cancer of that organ. I have listened to the statistics from the gentleman who has already occupied your attention, with great interest and with great pleasure, because, in almost every point, while they have not corresponded with published statistics as we now have them, they have corresponded with my own. In four of these thirteen cases in which I operated for cancer of the breast, I know nothing of the results. Two of the thirteen cases are still living. All of the seven remaining cases died at periods varying from eighteen months to four years after the operation. A curious point in relation to them was, that the one who lived the longest—and this point I have not seen alluded to by any author—was the patient who was the oldest. That patient was 71 years of age when I operated, and had been afflicted with the disease some four or five months when I first saw her.

There was no apparent return of the disease until several months afterwards, and then there was probably a return of the disease to some internal organ. The point is this: whether the progress of pathological changes is not exactly in the same ratio as the metamorphosis of tissue in relation to age; whether in persons of advanced life we may not account in this way for the longer exemption from a fatal termination of the disease than when the disease occurs in those who are less advanced in age.

In 1858, although I had refused to have anything to do with general surgery, and confined my operations entirely to the obstetrical department, I had one patient who ab olutely refused to permit any one else to operate upon her except myself. I accordingly removed her breast. The axillary glands were not involved, but the disease returned within a very few months, and the patient died eleven months after the operation.

The second case which I will refer to is a rather curious and rather exceptional one. It occured in the year 1860, in a lady 43 years of age, and she had the disease for several months when I first saw her, and in what I regarded as a very malignant form. That person, again, utterly refused to have an operation performed unless I would perform it myself, and I accordingly performed the operation, assisted by Dr. Foster Swift and Dr. Charles Phelps. In that case acupressure was employed, as I believe, for the first time in this city, and I was very much interested and pleased with the effect of acupressure in diminishing the amount of suppuration, which in that case was very slight indeed. The patient was operated upon in April, 1860. In my own belief, and in the belief of the microscopist, it was one of the most malignant forms of this disease of the breast. and yet the woman was alive in 1871. I simply mention this case as a small contribution to the number of successful operations in the sense of curative, in cases of carcinoma of the breast. That specimen was afterwards presented at the New York Pathological Society, and the minutes of the meeting, which were published in the *Medical Record*, represented it as being presented by Dr. Swift and that the operation had been performed by Dr. Parker, which is a fair illustration of the uncertainty of surgical glory. With regard to statistics in determining whether a surgical operation shall be performed or not, most modern writers agree that operations do, in a certain proportion of cases which are judiciously selected, absolutely and positively prolong life, relieve suffering, and in some cases actually save life. The diametrical opposition which the statistics of some surgeons have to those of other surgeons who are equally well situated for making observations, may perhaps be explained in this way. One surgeon may be of the opinion that the disease is, primarily, always a local disease, and that its constitutional character is secondary to the local disease, which manifests itself differently in different cases. If this theory be correct, the proper method of treatment is the early extirpation of all suspicious-looking growths. On the other hand, other surgeons are of the opinion that the disease is a constitutional disease; that operations are deleterious in their effects, and should not be resorted to until all other means have failed to arrest its progress.

Again, some surgeons who have a greater fondness for operations than others, will remove a suspicious-looking growth much earlier than those surgeons who are less fond of operations, so that in some cases it may be that the delay in the performance of the operation has permitted the disease to make such extensive ravages upon the general system, that the operation, if performed at all can be p rformed with the expectation of giving some relief from distressing symptoms.

I began in early life as a m st enthusiastic believer in the numerical system, regarding it as a most efficient means for advancing our knowledge of disease. But my experience has proven to me that statistics which ordinarily receive publication are extremely unreliable, and that they form a most unstable foundation upon which to predict future action, whether it shall be for the formation of an opinion or made the basis of an operation. The statistics which the author of the paper has given us relative to the comparative frequency of cancer of the breast singularly accord with the statistics from the cancer hospitals in the city of London. Out of 7,800 cases which were under treatment in that city between the years 1851 and 1861, 4,388 were cancer of the breast. This is from an entirely different sphere of observation, and yet the result of the observation shows that the female breast is one of the most favorite places in the human body for the development of this disease. It seems to have an elective affinity for the female breast, and perhaps in the progress of etiology and the science of physiology the reason for this elective affinity will be discovered.

The next point which I will notice in connection with the paper, is with regard to hereditary predisposition to the disease. I feel quite confident that I should never have read a paper which I did read, and which was published by the Academy of Medicine, upon "The Clinical Study of Cancer of the Uterus," had I not been thoroughly convinced upon this point. When I came to study my own observations, I found that some of them were so different from the published statements in published works that I felt doubtful about reading them without consultation with some of my personal friends. My own statistics with regard to hereditary predisposition to cancer of the uterus almost exactly correspond to the observations of the author of this paper with regard to hereditary predisposition to cancer of the female breast.

Another very interesting point to me was, that the author of the paper has found so much larger proportion of cases of cancer of the breast where hereditary predisposition to cancer was entirely absent, but where hereditary predisposition to tubercles was present. The results of his observations upon this point give the same relations which are found in my own statistics, and I believe that the idea of hereditary predisposition to cancer should be denounced, and that this denunciation should be pronounced boldly by physicians.

There were a few points to which no allusions were made, and concerning which I wish to make some inquiry

What is meant by a cancerous cachexia? In my earlier experience I was always looking for something like a cancerous cachexia, but my later experience and observation have taught me to become a non-believer, and I do not now believe at all in cancerous cachexia, as the term is commonly used. I have seen patients in the most advanced stages of cancer of the uterus, and in almost all its various phases, when they presented the appearance of robust health. The cachexia, when it does appear, is to my mind not a measure of the influence which has been produced by the simple presence of cancer in the system, but rather from associated lesions of the various organs of the body.

These are my observations with regard to cancer of the uterus, and I should like to know whether the same thing has been observed with regard to cancer of the breast.

Another point, which was not alluded to, and concerning which I should be pleased to gain some information, is, with regard to the value of pain as a symptom in cancer. I am of the opinion that it is a symptom of uncertain value in aiding us in determining the existence or non-existence of cancer of the uterus. I have seen patients in the advanced stages of the disease without the slightest suspicion having been raised with reference to the presence of the disease by any pain. My own opinion is, that pain is simply a measure of the influence which the disease has had upon the contiguous and adjacent tissues. Cancer may occur so as to interfere with the functions of the uterus, or affect the subperitoneal tissues; and when these tissues are affected we are sure to have pain, and in some of these cases the pain is most atrocious. In other cases, where the disease presents more malignancy, the pain is sometimes very trivial. Whether the amount of pain is in relation to the amount of influence which the disease has upon the adjacent and contiguous tissues, I am not able to say, but simply throw it out as a question for consideration.

From time immemorial there has been an attempt made to destroy cancer by the use of every variety of known caustics. It has been a favorits resort of empiricism, and the most successful and perhaps the most lucrative of all charlatanism has been seen in the use of caustic agents to destroy the local manifestations of cancer. As a consequence of this, of course, a great majority of the surgical world have been satisfied with regard to the uselessness of such attempts. My own prejudices have always been against this method of treatment. I once attempted to make some observations respecting this plan of treatment as it was then adopted in St. Bartholomew's Hospital, and the whole process was so revolting that I did not pursue my investigations farther, and the result of my observations was not at all favorable.

In the year 1870, however, I was consulted by a lady who had a tumor in the breast which was very suspicious in its character, and which I watched for some weeks, when I regarded it as cancer, and urged upon my patient the importance of having it removed at once. But the patient utterly refused to have any cutting operation performed. At that time I had been studying up the subject somewhat, and among other works which I had read was Marsden's work upon the use of caustics in the treatment of cancer.

The same summer, while abroad, I visited the hospital in which Dr. Marsden had made his observations and applied his treatment, and saw the results of this treatment. I became so much interested in this plan of treatment and was so highly pleased with it, that, upon my return, I recommended to my patient to submit to the treatment by the use of caustics. After some delays she consented. The form of cancer from which she was suffering was apparently of the most malignant type, and at the time I commenced the treatment the mass was about two inches in diameter, which is the extreme limit in size permissible to be treated by this method. In the course of eighteen days after the first application was made, the mass came away, the process of cicatrization was completed in a short time, and there has not been the slightest appearance of return up to this time.

Another case to which I wish to make reference, was in a patient who had had two sisters die with cancer of the breast, but her father and mother were still living at the time she consulted me. Not the slightest suspicion of cancer could be traced in either member of the family One sister died some six or seven years ago from cancer of the breast. The other sister I was called to visit, and I found the axillary glands involved in the disease; there were evidences of what is known as the cancerous cachexia, and I called for council. Dr. Van Buren was called in consultation, but the case was regarded as utterly hopeless, and the patient died without an operation.

The third sister came under my observation for epithelioma of the uterus. That patient I operated upon in 1866, removing the cervix uteri by amputation. It is now seven years since the operation was performed, and she remains in the most perfect health.

About five years ago a lady consulted me with regard to a suspicious-looking tumor in her right breast. She was under my observation for about two years, and received treatment, but I never was of the opinion that the growth was malignant. At the end of two years it entirely disappeared. In February, 1873, that patient came back to me with a tumor in her left breast, which I regarded as true cancer of the breast. The tumor had been observed for more than a year, and when I saw it, the nature of the case seemed clear and positive. Its removal was recommended. Consultation was held, to satisfy the patient with regard to its nanature, the propriety of its removal, and if decided to remove it, how it should be removed. It was decided to remove the tumor by Marsden's treatment, and the treatment was accordingly commenced upon the first day of April. The amount of pain which the patient has suffered during the course of the treatment has been very insignificant indeed. She has been up most of the time, has been able to be out riding some of the time, and it is now eighteen days since the first application, and the slough is just ready to come away. The treatment of this case thus far has been very pleasant. What the result of the case may be it is impossible at present to decide.

I will now describe the plan of treatment as given by Dr. Marsden—the plan which he professes to have derived great success from, not only in a very considerable number of cases of cancer of the breast, but in the treatment of cancer of various parts of the body, and even of cancer of the neck of the uterus.

This method of treatment is limited to cases in which the surface of the tumor does not extend over two inches. Care must be taken that the paste is of sufficient consistence so as not to flow beyond the point to which it is applied. The general formula for the preparation of the caustic is to combine arsenious acid and mucilage in such quantities as to make a thick paste, and the formula commonly employed for this purpose is—

℞ Arsenious Acid, - - - - dr. ij.
Mucilage, - - - - - - dr. j.

This paste is spread over the surface of the tumor, and two or three layers of lint spread over that. The lint absorbs all the surplus paste and protects from farther cauterization. The first application is left on for twenty-four or forty-eight hours, according to the extent of surface, and then removed by gently soaking it with warm water After the old paste has been removed in this way, one judges from the impression made with regard to a farther application of the caustic. These applications are to be continued until a line of demarcation entirely surrounding the deceased structure is shown. Then the lint is soaked and removed, and a bread-and-water poultice applied, and changed every few hours. At first there is sometimes considerable inflammatory action set up, but the amount of pain is very inconsiderable as compared with the use of the knife, and the process of cicatrization is equally painless and satisfactory.

The shock to the system, as a rule, is very much less. The constitutional effect of the arsenic in this case was very slight, lasting only a few hours, and then passed away. Indeed, the moderate constitutional effect of arsenic I have long believed to have a certain positiveness in the treatment of cancer, in that it retards the proliferation of cancerous tissue. I mention these cases with the hope that it may contribute something to our knowledge of means by which we may meet this most terrific disease.—*Med. Record*

